# Validation of the Multi-INdependence Dimensions (MIND) questionnaire for prolonged mechanically ventilated subjects

**DOI:** 10.1186/s12890-019-0870-2

**Published:** 2019-06-20

**Authors:** Joao C. Winck, Hélène Gilet, Peter Kalin, Javier Murcia, Fabian Plano, Antoine Regnault, Michael Dreher, Michele Vitacca, Nicolino Ambrosino

**Affiliations:** 1 Linde AG, Linde Healthcare, Pullach, Germany; 2 Patient-Centered Outcomes Mapi, Lyon, France; 3 Linde Gas Therapeutics GmbH, Linde Healthcare, Oberschleissheim, Germany; 4 Division Homecare Linde Healthcare, Bogota, Colombia; 5 REMEO Center Pilar, Linde Group, Buenos Aires, Argentina; 6 Division of Pneumology, Angiology and Intensive Care Medicine, University Hospital RWTH Aachen, Aachen, Germany; 7 Respiratory and Rehabilitation Division, ICS Maugeri, IRCCS, Lumezzane, Italy; 8 Fondazione Volterra Ricerche, Volterra, Italy; 9 Present Address : Faculty of Medicine, University of Porto, Porto, Portugal

**Keywords:** Prolonged mechanical ventilation, MIND questionnaire, Questionnaire validation, Composite scores, Rehabilitation, Health status assessment

## Abstract

**Background:**

Evaluating severity of illness of patients with prolonged mechanical ventilation (PMV) is important to adopt the best appropriate care management for each individual. Yet, no severity-of-illness scoring system has been specifically designed for this type of patients. The aim of this study was to develop and validate a new instrument, the Multi-INdependence Dimensions (MIND) questionnaire designed to comprehensively measure the severity of illness of patients under PMV.

**Methods:**

The validation of the MIND questionnaire was performed during a longitudinal observational study conducted with PMV subjects in weaning facilities in three countries (Argentina, Colombia and Germany). The questionnaire validity was tested in 3 stages: 1) Specification of components, with description of item responses, inter-item and Cronbach alpha correlations; 2) Creation of the composite scores; 3) Measurement properties determination including test-retest reliability after 30 days, clinical validity (Medical Research Council (MRC) muscle strength score, Sepsis-related Organ Failure Assessment (SOFA), Glasgow Coma Scale (GCS), Dependence Nursing Scale and EuroQol-5 Dimension evaluated at inclusion), and ability to detect change.

**Results:**

A total of 128 subjects participated in the validation study. Eleven component scores and four composite scores were created. MIND scores significantly correlated with MRC muscle strength, SOFA, DNS, GCS and EQ-5D, supporting the validity of the new scores. Intraclass Correlation Coefficient greater than 0.82 were observed for all composite scores, indicating good test-retest reliability. MIND scores were able to detect improvement in subject severity of illness.

**Conclusion:**

The MIND questionnaire is a valid and reliable instrument for measuring comprehensively the multiple dimensions characterizing the severity of illness of PMV patients.

**Trial registration:**

NCT02255058.

**Electronic supplementary material:**

The online version of this article (10.1186/s12890-019-0870-2) contains supplementary material, which is available to authorized users.

## Background

According to the National Association for Medical Direction of Respiratory Care (NAMDRC) consensus conference, prolonged mechanical ventilation (PMV) refers to patients who require at least 6 h of mechanical ventilation for more than 21 consecutive days [1]. Recent estimates indicate that in the US the numbers of patients on PMV are expected to double by the year 2020, reaching more than 600,000 patients [2].

PMV patients, also referred to as chronically critically ill patients [3], are very demanding in terms of healthcare needs due to multiple systems and organs dysfunctions. Beyond prolonged dependence on mechanical ventilation, muscle, neuro-endocrine, skin and brain dysfunctions accumulate into a distinct and complex syndrome impacting not only families of the chronically critically ill patient but also the whole health care system [3]. Evaluating the health status of these patients is of major importance to adopt the best appropriate care management and better define prognosis by better understanding their general health, and to evaluate results of multidisciplinary rehabilitation [4].

The commonly used severity-of-illness scoring systems like the Sepsis-related Organ Failure Assessment (SOFA) score or the Acute Physiology, Age, Chronic Health Evaluation III were developed in acute intensive care unit (ICU) population [5]. However, these systems were not developed nor validated in PMV patients and as a result caution should be used when using them in this population [6]. The Functional Independence Measure (FIM), one of the most widely used scoring systems, was developed to standardise assessment of functional status during medical rehabilitation [7]. However, the FIM is mainly focused on the motor and cognitive dimensions, and validation was demonstrated mainly in stroke patients with only 1% of the population suffering from pulmonary impairment [8]. Few studies including small numbers of patients have used the FIM in the context of PMV [9–12]. In addition, the FIM omits important dimensions such as the respiratory function, sleep and co-morbidity. More recently, multi-factorial scores have been developed, like the Chelsea Critical Care Physical Assessment Tool (CPAx) that extends evaluation to cough ability and ventilator dependence [13], or the Burns Wean Assessment Program (BWAP) [14] with an extensive respiratory assessment. However they also were only tested in the ICU setting with non-PMV patients. Therefore, the development of adequately performing health status, from the severity of illness and dependence angle in particular, measures appropriate to this specific patient population is needed.

We hypothesized that an originally created multidimensional comprehensive score would objectively evaluate severity of illness of PMV subjects.

The Multi-INdependence Dimensions (MIND) questionnaire has been developed as a comprehensive score specifically designed to assess the health status for PMV patients and covers areas allowing the evaluation of the severity of illness and dependence of PMV patients. It includes Cognition, Feeding/swallowing, Sleep, Skin integrity, Oxygenation, Cough strength, Secretion management, Ventilator dependence, Mobility, Upper limb and lower limb strength and Co-morbidities. The objective of this study was to assess the measurement properties of the MIND questionnaire.

## Methods

### Development of the MIND questionnaire: qualitative phase

For the first developmental step, a literature search was performed to identify the existing instruments already used in critical care and the components relevant in respiratory care and rehabilitation, in the PMV setting. Then, the Delphi method was applied including four participating experts (JCW, MD, MV and NA). The different Delphi rounds aimed at ranking the identified components from what the specialists thought was the most important to the least important to define severity of patients under PMV. The items were initially divided into 3 parts: A) Functional tests, Quality of Life, Activities of Daily Living; B) Co-morbidities, Neurocognitive evaluation; C) Nutrition, mental status, sore risk. Also the panellists decided it was very important to include respiratory items (like oxygenation, ventilatory dependence and cough strength). Concerning part A, from 12 functional tests 8 were chosen; in relation to part B, from 12 Comorbidity and Neurocognitive tests 4 were selected; and regarding Part C, from 8 Nutrition, mental status and sore risk tests, 3 were selected. Selection was based on the application on critical care or PMV patients. Each of the panellists chose 10 very important items and 10 not so important items (from the list of 91), Opinions expressed during one round of the questionnaire were returned to the group during the next round in the form of statistical summaries. After 3 rounds, items were then developed for each of the components identified and last agreed upon as key disability-related components specific to patients under PMV. Cognitive interviews were conducted with doctors (*n* = 2), nurses (*n* = 4) and therapists (n = 4) to test the content validity, clarity and ease of use of the newly developed questionnaire. Finally a pilot study was performed in 2 centres (involving 2 doctors and 2 therapists) including 30 patients (20 in 2 weaning centres and 10 in a respiratory rehabilitation program), to test the use of the questionnaire in real conditions. The questionnaire was revised based on the health profesionnals’s feedback. The resulting MIND questionnaire was composed of 18 items grouped into 11 components: Cognition (2 items), Feeding/swallowing (1 item), Sleep (5 items), Skin integrity (1 item), Oxygenation (1 item), Cough strength (1 item), Secretion management (1 item), Mobility (2 items), Upper and lower limb strength (2 items), Ventilator dependence (1 item) and Co-morbidities (1 item). The questionnaire is described in Table 1 and shown in a Additional file 1. All items are 6-point ordinal scales, with higher values (score 5) indicating best outcome/normal functioning, and lower values (score 0) indicating worst outcome/inability to function. The MIND questionnaire is to be completed by three different healthcare professionals: medical doctors, nurses and physiotherapists. The manual for the evaluation of the items is included in a Additional file 2.

**Table 1:** Structure and item description of the MIND Questionnaire

Component	Number of items	Item Description
Cognition	2	Orientation/Speech
Feeding/swallowing	1	Feeding/swallowing
Sleep	5	Sleep depth Sleep latency Awakenings Return to sleep Sleep quality
Skin integrity	1	Skin integrity
Oxygenation	1	Oxygenation
Cough Strength	1	Cough Strength
Secretion Management	1	Secretion Management
Mobility	2	Sit to stand/ Stand to sit
Upper limb strength /Lower limb strength	2	Upper limb strength Lower limb strength
Ventilator Dependence	1	Ventilator Dependence
Co-morbidities	1	Co-morbidities from the modified Charlson index list

*MIND* Multi-INdependence Dimensions

### Validation of the MIND questionnaire: quantitative phase

#### Study subjects

Eligible subjects had to be aged between 18 and 80 years, with a diagnosis of acute lung injury, chronic obstructive pulmonary disease (COPD)/chronic lung disease, neuromuscular disorders, post-operative, cardiovascular disorders, trauma, or other. They (or the family member or legally responsible person in cases where subjects whose health condition did not allow them to answer personally) had to provide written informed consent; and be able to read and understand the study procedures and comply with the requirements of the study.

Subjects with expected length of stay of less than 48 h or unable to achieve consent were not included.

#### Study design

This longitudinal observational study was conducted in five REMEO® centres from Germany (Berlin and Mahlow), Colombia (Bogota and Medellin) and Argentina (El Pilar) belonging to an international network of facilities dedicated to weaning and management of post-ICU subjects. The REMEO® centres included in this study are close to a long-term acute care facility. REMEO® services are provided by multi-disciplinary teams, specialised and experienced in caring for patients with prolonged mechanical ventilation needs. These teams cover a full spectrum of clinical and medical care (basic to advanced nursing care, including respiratory therapy and comprehensive rehabilitation therapy); the ratio of nurse/patient is 1:4 (http://www.remeo.com/en/index.html).

All existing insubjects or subjects admitted within the sampling frame and who met inclusion criteria were recruited. At inclusion, demographic and clinical characteristics were collected. Healthcare professionals trained on the study completed the MIND questionnaire and the following other outcome measures: Medical Research Council (MRC) score for quadriceps and biceps strength [15], SOFA score [5], Glasgow coma scale (GCS) [16] and Dependence Nursing Scale (DNS) [17]; subjects were asked to complete the EQ-5D [18]. These tools were chosen because they had already been used in studies including PMV subjects and were considered easy to apply by the investigators [19–22].

At one day and 30 days after inclusion both the MIND questionnaire and Clinician Global Impression of Change (CGIC) were collected. These outcome measures were interpreted as follows: MRC scales ranged from 0 to 5 (superior muscle strength) [15]; SOFA ranged from 0 to 24 (higher degree of organ dysfunction) [5]; GCS ranged from 3 to 15 (better behavioural responsiveness) [16]; DNS score ranged from 0 to 43 (higher level of dependence) [17]; Katz activity of daily living index ranged from 0 to 6 (greater level of independence) [23]; CGIC was a 5-point Likert scale ranging from “Much worse” to “Much better”. The EQ-5D included 5 items measuring Mobility, Self-care, Usual activities, Pain/Discomfort and Anxiety/Depression and a visual analogue scale (VAS) ranging from 0 (worst imaginable health state) to 100 (best imaginable health state).

#### Analysis

The analysis set included all subjects who met all inclusion criteria. All analyses were performed using SAS v9.2 or later (SAS Institute; Cary, NJ, USA).

Specific areas of health status are referred to as components and the combination of areas into an overall assessment of health is referred to as a composite score in line with Bollen et al. [24]. Composite scores are particular in that their components need not be related and are thus not necessarily highly correlated [25].

**Fig. 1:**
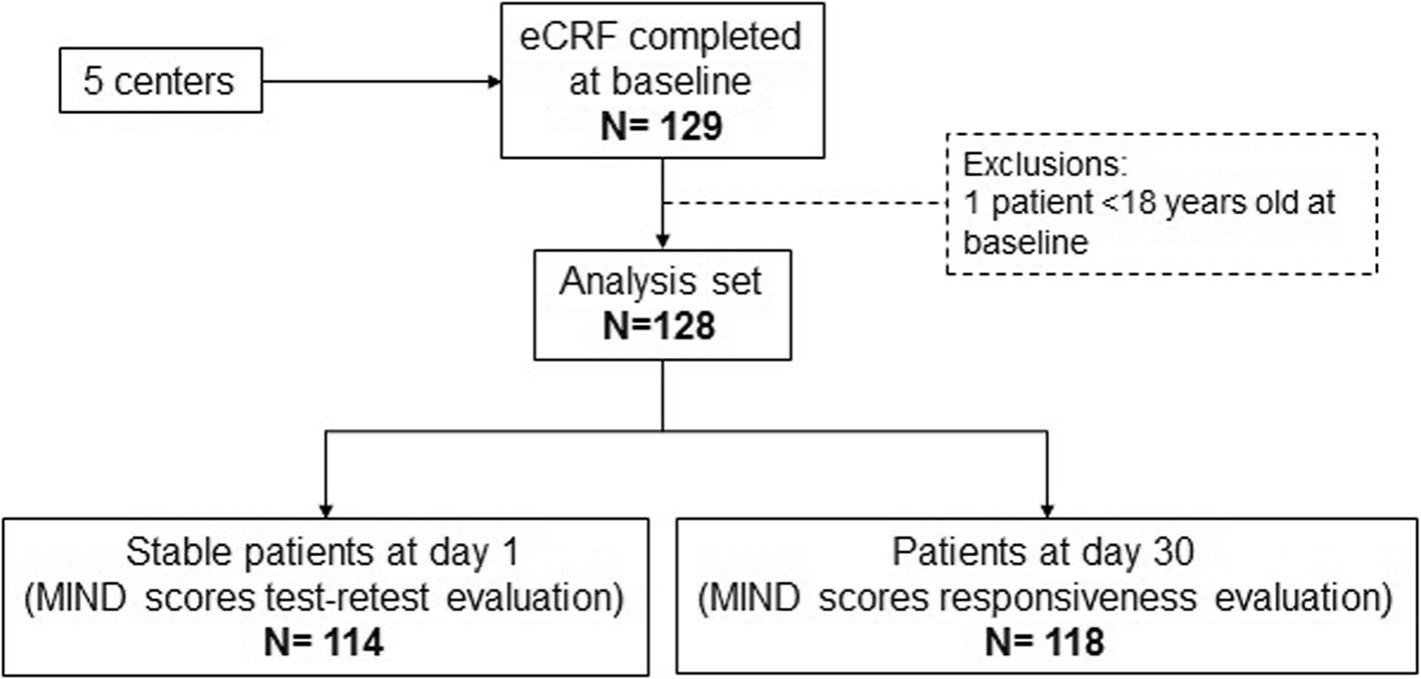
Analysis strategy

The validation process of the MIND questionnaire was adapted accordingly and consisted of the three following stages (Fig. 1). The objective of the component specification stage was to identify issues with item response distributions or item redundancy within a component. Analyses consisted of descriptions of item responses (description of a floor or ceiling effect) and, for multi-item components, internal consistency reliability using Cronbach’s alpha coefficient [26] and Pearson correlation coefficients to measure association between items of a component. MIND component scores were calculated as the mean of all items within a component. The objective of the composite score creation stage was to combine components into a single score. Both simple summation and weighted summation of components scores were tested in an attempt to optimise the measure and facilitate comparison and interpretation of the results [25]. Weighting was performed using univariable linear regression to link MIND components to the EQ-5D VAS, which was chosen as an informative measure of subject general health. A short-form MIND score was explored using only components which were significantly associated with the EQ-5D VAS resulting from a multivariable regression model retaining components (*p* < 0.05) using a stepwise selection procedure. The final stage aimed to investigate the measurement properties of these scores (reliability, validity, and ability to detect change). Test-retest reliability of MIND components and composite scores was assessed by calculating Intraclass Correlation Coefficients between assessments at baseline and day 1 for subjects who were considered to have not changed as measured by the CGIC. Construct validity was assessed by estimating associations with other outcome measures included in the study (MRC scores, SOFA, GCS, DNS, EQ-5D VAS). For categorical outcomes the distribution of MIND scores were described per category, and for continuous outcomes, Pearson correlation coefficients were calculated. Ability to detect change was assessed by calculating effect sizes (ES) of the change in score between baseline and 30 days in subjects who respectively improved, did not change and worsened according to the CGIC [27].

The measurement properties of the different composite scores were compared to select a final composite score. All decisions regarding composite score development were considered from both statistical and clinical perspectives.

## Results

### Subject description

**Fig. 2:**
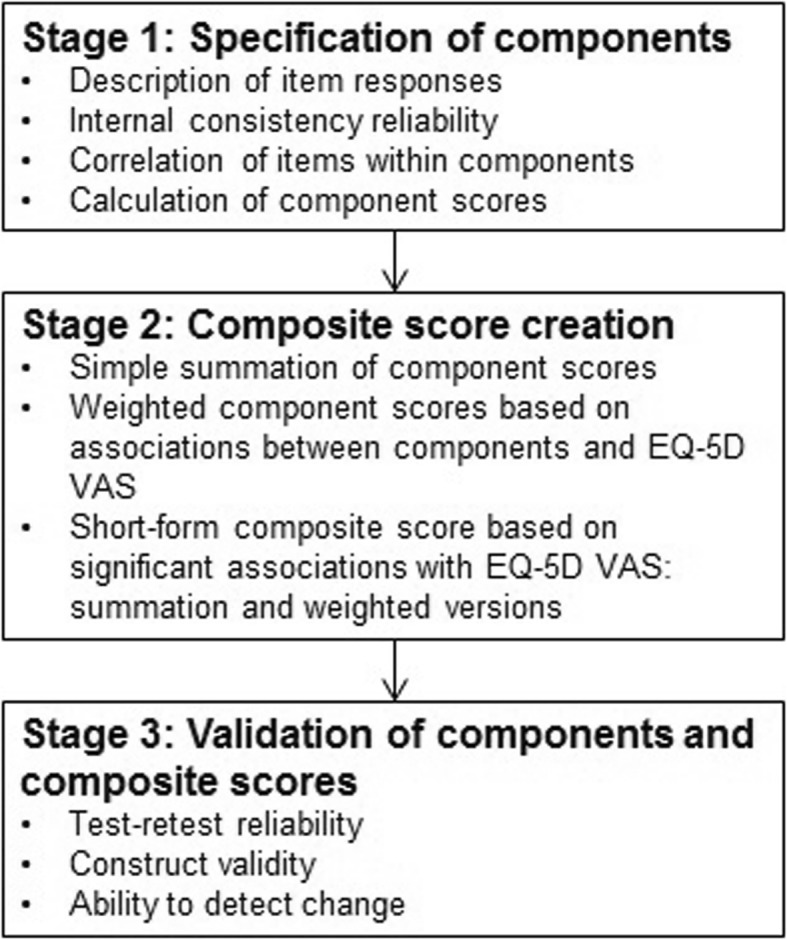
Study flow chart

All 129 recruited subjects but one met inclusion criteria (Fig. 2). Among those 128 subjects composing the analysis set, 114 were considered stable after 1 day; stability meant the patients were hemodynamically stable, with ventilation and oxygenation stability, afebrile, no chest radiograph abnormalities and no change of treatment plan; 118 had an assessment at 30 days. About a third of subjects were diagnosed with COPD/Chronic lung disease (Table 2). The vast majority of subjects were tracheostomized (93.7%), many requiring complete ventilator dependence (48.0%), undergoing very frequent suctioning (50.8%), unable to stand (51.0%), having 2 or more co-morbidities and 46.0% of them needing a gastrostomy tube. Among the 110 subjects who completed the EQ-5D at baseline, about half reported being confined to bed, unable to wash or dress themselves, and unable to perform their usual activities (Additional file 3: Table S1).

**Table 2:** Patients demographic and clinical characteristics (*N* = 128)

Time of assessment	Characteristics		Total (*N* = 128)
Baseline	Age (years) – *N* = 128	Mean (SD)	65.0 (16.5)
		Min – Max	18.0–89.0
	Gender (%) – *N* = 128	Male	50.0
	Previous days of mechanical ventilation (days) – *N* = 111	Mean (SD)	78.1 (134.9)
		Min – Max	11.0–1025.0
		Median	46.0
		Q1 – Q3	32.0–71.0
	Primary diagnosis (%) – *N* = 128	Post-Acute Lung Injury	6.3
		COPD/Chronic Lung Disease	34.4
		Neuromuscular Disorders	18.8
		Post-Operative	8.6
		Cardiovascular Disorders	9.4
		Trauma (Spinal Cord and Head Injury)	5.5
		Cerebrovascular Disorders	10.9
		Other	6.3
	Use of (%) – *N* = 128	Non-invasive ventilation	6.3
		Invasive ventilation	90.6
		Tracheostomy collar	3.1
	MRC scale quadriceps (%) – *N* = 128	0–2	48.4
		3	20.3
		4–5	28.9
		Missing	2.3
	MRC scale biceps (%) – *N* = 128	0–2	35.9
		3	27.3
		4–5	34.4
		Missing	2.3
	SOFA score (%) – *N* = 128	0–2	83.6
		3–8	16.4
	GCS score – N = 128	1–8	9.4
		9–12	13.3
		13–15	77.3
	DNS score – *N* = 128	Mean (SD)	20.4 (6.3)
		Min – Max	3.0–32.0
	EQ-5D VAS – *N* = 110	Mean (SD)	47.5 (18.1)
		Min – Max	10.0–100.0
Day 30	CGIC (%) – *N* = 128	Much worse	6.3
		Worse	6.3
		Same status	42.2
		Better	25.8
		Much better	11.7
		Missing	7.8

*SD* Standard deviation, *MRC* Medical Research Council, *SOFA* Sepsis-related Organ Failure Assessment, *GCS* Glasgow Coma Scale, *DNS* Dependence Nursing Scale, *EQ-5D VAS* EuroQol-5 Dimension visual analogue scale, *CGIC* Clinician Global Impression of Change

### Validation of the MIND questionnaire

#### Component specification stage

**Fig. 3:**
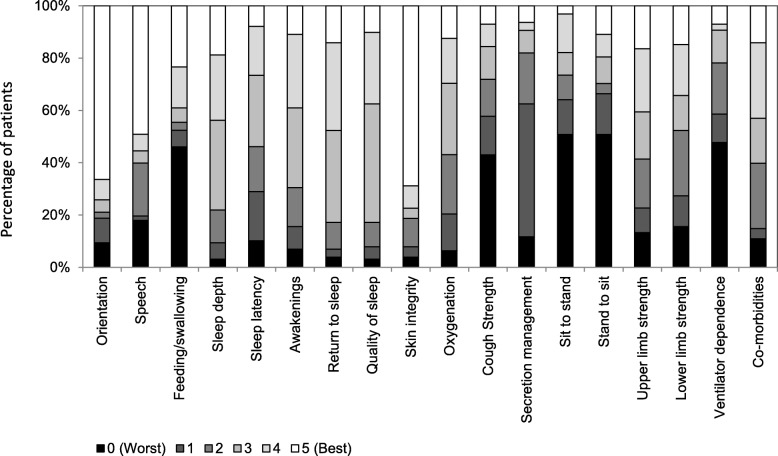
Responses to MIND items at baseline (*N* = 128)

At baseline, a substantial percentage of subjects scored the worst response choice for some MIND items (Fig. 3) indicating potential floor effects: Feeding/swallowing (46%), Cough strength (43%), Sit to stand (51%), Stand to sit (51%) and Ventilator dependence (48%). The MIND questionnaire took 10–15 min for HCPs to complete and there were no missing responses to any of the MIND items at any of the three time-points. Internal measurement properties for the Cognition and Upper limb and lower limb strength components of the MIND questionnaire were supported by high inter-item correlations and good internal consistency reliability (Table 3). The two items of the Mobility component were very highly correlated (correlation coefficients = 0.98) indicating item redundancy, also confirmed by Cronbach’s alpha (0.99). Accordingly, the “Stand to sit” item was removed. In the Sleep component, the “Sleep latency” item was found to be weakly correlated with the other items (− 0.16 to 0.00) and decreased Cronbach’s alpha (from 0.90 to 0.72) and was thus removed. The resulting 4-item Sleep component had good internal consistency with inter-item correlations of 0.55 to 0.78 and Cronbach’s alpha of 0.90. High correlations between components were characterised between the Upper limb and lower limb strength, Cognition, Feeding/swallowing and Mobility components (correlations 0.47–0.75); and the Ventilator dependence, Cough strength and Secretion management components (correlations 0.45–0.55) (Table 4). All other inter-component correlations were smaller than 0.30.

**Table 3:** Specification of multi-item MIND component scores and creation of MIND composite score (N = 128)

Component	Initial Number of items	Items	Multi-item component scores			11-component composite score		2-component composite score	
			Range of inter-item correlations^a^	Cronbach’s α^b^		Weights for simple summation	Weights for weighted summation^c^	Weights for simple summation	Weights for weighted summation^c^
				Overall	With item removed				
Cognition	2	Orientation Speech	0.70	0.82	–	1	3.4		
Feeding/swallowing	1	Feeding/swallowing				1	2.8	1	2.8
Sleep	5	Sleep depth Sleep latency Awakenings Return to sleep Quality of sleep	−0.16 – 0.78	0.72	0.61 0.90 0.57 0.58 0.56	1	3.7		
Skin integrity	1	Skin integrity				1	1.0		
Oxygenation	1	Oxygenation				1	−1.8		
Cough strength	1	Cough strength				1	3.5	1	3.5
Secretion management	1	Secretion management				1	0.9		
Mobility	2	Sit to stand Stand to sit	0.98	0.99	–	1	3.2		
Upper limb and lower limb strength	2	Upper limb strength Lower limb strength	0.89	0.94	–	1	2.8		
Ventilator dependence	1	Ventilator dependence				1	1.2		
Co-morbidities	1	Co-morbidities				1	0.3		

^a^Pearson coefficient

^b^Recommended threshold: Cronbach’s alpha > 0.70

^c^From univariate regression

**Table 4:** Inter-MIND component correlations^a^ at baseline (*N* = 128)

Component	Cognition	Feeding/ swallowing	Sleep	Skin integrity	Oxygenation	Cough strength	Secretion management	Mobility	Upper limb and lower limb strength	Ventilator dependence	Co-morbidities
Cognition	1.00										
Feeding/swallowing	**0.60**	1.00									
Sleep	0.09	0.17	1.00								
Skin integrity	−0.02	0.14	0.02	1.00							
Oxygenation	0.00	−0.14	0.12	− 0.03	1.00						
Cough strength	0.11	− 0.02	0.07	0.01	0.07	1.00					
Secretion management	0.06	−0.03	0.15	− 0.04	0.30	**0.48**	1.00				
Mobility	**0.47**	**0.68**	0.11	0.26	−0.12	−0.01	− 0.02	1.00			
Upper limb and lower limb strength	**0.59**	**0.66**	0.02	0.15	−0.13	0.05	−0.02	**0.75**	1.00		
Ventilator dependence	0.17	0.15	0.11	0.03	0.18	**0.45**	**0.55**	0.19	0.20	1.00	
Co-morbidities	0.00	−0.09	0.17	−0.12	0.17	0.17	0.32	−0.15	−0.29	0.27	1.00

^a^Pearson correlation coefficient

In bold, correlation > 0.40

#### Composite score creation

Four composite scores were created (Table 3): two scores including all 11 components, one corresponding to a simple summation and one to weighted scores; and two short-form scores including only the components Feeding/swallowing and Cough strength: one corresponding to a simple summation and one to weighted scores.

#### Measurement properties of the composite scores

**Fig. 4:**
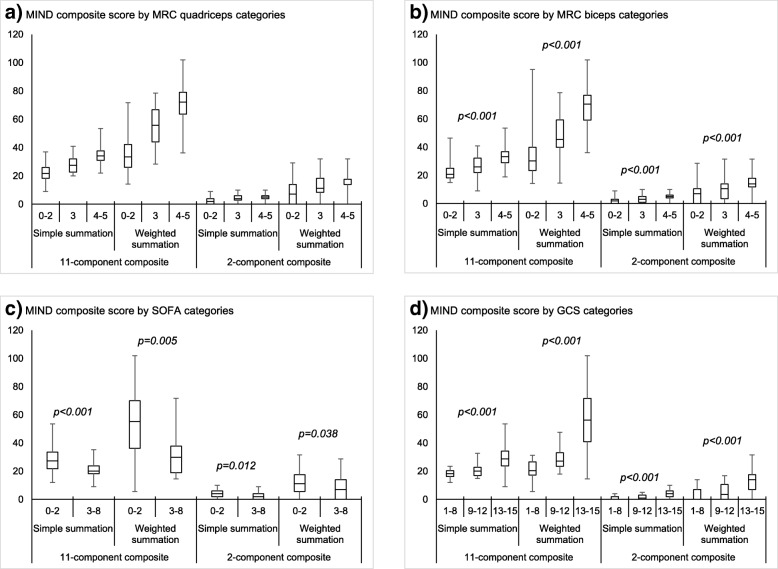
Comparison of MIND composite scores at baseline according to MRC scores, SOFA score and GCS (*N* = 128)

All four composite scores showed good test-retest reliability in subjects with a stable health status from baseline to Day 1 (ICC ranging from 0.67 to 0.92 for all components, except Sleep (ICC = 0.38) and Ventilator dependence (ICC = 0.51), and construct validity (Table 5). All four composite scores were able to significantly discriminate subjects according to MRC scales quadriceps and biceps, SOFA score and GCS, with higher MIND scores for less severe MRC, SOFA and GCS scores (Fig. 4 and Additional file 4: Table S2). Finally, statistically significant differences in changes in MIND scores were observed between subjects considered worse, same status or better after 30 days compared to baseline; with moderate-large ES for subjects who had improved (Table 5).

**Table 5:** Validation (test-retest reliability, clinical validity and ability to detect changes) of MIND components and composite scores (*N* = 128)

MIND score		Final Number of items	ICC^a^ (*N* = 114)	Correlations with external measuresb		Comparison of changes in scores from baseline to day 30 – Mean change in score (ES^c^)			
				DNS score (*N* = 128)	EQ-5D VAS (*N* = 110)	CGIC = worse (*N* = 16)	CGIC = stable (*N* = 54)	CGIC = better (*N* = 48)	*p*-value^§^
Component	Cognition	2	0.89	− 0.50	0.31	− 0.5 (− 0.4)	0.1 (0.1)	0.5 (0.3)	< 0.001
	Feeding/swallowing	1	0.83	−0.57	0.34	0.1 (0.0)	0.1 (0.0)	1.0 (0.5)	< 0.001
	Sleep	4	0.38	−0.23	0.21	0.6 (0.5)	−0.2 (− 0.2)	0.4 (0.4)	0.011
	Skin integrity	1	0.84	−0.35	0.07	−0.1 (− 0.0)	0.1 (0.1)	0.2 (0.1)	0.519
	Oxygenation	1	0.67	−0.03	− 0.13	−0.3 (− 0.2)	0.2 (0.2)	0.5 (0.3)	0.119
	Cough strength	1	0.71	−0.20	0.32	−0.3 (− 0.2)	0.0 (0.0)	0.3 (0.1)	0.415
	Secretion management	1	0.70	−0.27	0.06	−0.3 (− 0.2)	−0.1 (− 0.1)	0.8 (0.5)	< 0.001
	Mobility	1	0.83	−0.61	0.29	−0.1 (− 0.1)	0.2 (0.1)	0.8 (0.5)	< 0.001
	Upper limb and lower limb strength	2	0.92	−0.46	0.25	−0.2 (− 0.2)	0.2 (0.1)	0.4 (0.3)	0.002
	Ventilator dependence	1	0.51	−0.50	0.10	0.2 (0.1)	0.1 (0.1)	1.6 (0.9)	< 0.001
	Co-morbidities	1	0.83	−0.16	0.03	0.1 (0.0)	0.1 (0.0)	−0.3 (− 0.2)	0.125
11-component composite	Simple summation	16	0.83	−0.76	0.38	−0.7 (− 0.1)	0.7 (0.1)	6.1 (0.7)	< 0.001
	Weighted summation	16	0.89	−0.71	0.46	−0.8 (− 0.1)	0.5 (0.0)	12.3 (0.6)	< 0.001
2-component composite	Simple summation	2	0.83	−0.58	0.46	−0.2 (− 0.1)	0.1 (0.0)	1.3 (0.5)	0.001
	Weighted summation	2	0.82	−0.56	0.47	−0.7 (− 0.1)	0.3 (0.0)	3.8 (0.5)	0.003

*ICC* Intraclass correlation coefficient, *ES* Effect size, *DNS* Dependence Nursing Scale, *EQ-5D VAS* EuroQol-5 Dimension Visual Analogue Scale; CGIC = Clinician Global Impression of Change

^a^Calculated from baseline to Day 1; Recommended threshold: ICC > 0.70

^**b**^Pearson correlation coefficient

^**c**^ES around 0.2: small change; ES around 0.50: moderate change; ES around 0.80: large change

^§^*p*-value from ANOVA to compare mean change in score between CGIC worse, stable and better groups

## Discussion

Our study aimed to develop and validate a new instrument to assess comprehensively the health status of prolonged ventilator dependent patients. The MIND questionnaire was originally designed based on clinical expert insight as a HCP-completed composite measure covering 11 key areas reflecting the health status of PMV patients. MIND scores were created using data from an international study and have shown to be valid and reliable in patients undergoing prolonged mechanical ventilation. To ascertain the role of this questionnaire as a tool to evaluate responses to treatment in PMV patients (like multidisciplinary rehabilitation), different severity of illness levels were included during the developement phase. The response categories of the questionnaire should thus cover and be adapted to the full range of responses from the very severe to the very mild.

The MIND questionnaire is novel in that it has been developed and validated in a PMV patient population and therefore is more suitable in contrast to other questionnaires that are currently in use in this setting such as CPAx [13], FIM [7], Physical Function ICU Test (PFIT-s) [28], Functional Status Score for the Intensive Care Unit (FSS-ICU) [29] that were designed for other patient populations and include either physical function domains (PFIT, FSS-ICU) or do not focus on all the affected domains of PMV patients (FIM, CPAx). The CPAx is the closest multi-factorial score system to the MIND questionnaire. The functional components of the CPAx focus on objective strength (dynamometer) while the MIND uses a system similar to the MRC muscle strength score; the respiratory function does not use ventilator dependency in hours as in the MIND and the cough assessment is more objective in the MIND that in the CPAx. Mobility is similarly measured in both scoring systems, but as a result of our expert review, secretion management, sleep and skin integrity were highlighted as important areas for PMV patients and integrated in the MIND questionnaire but are not covered by the CPAx. This reflects the fact that the CPAx was developed as a tool to be implemented by physiotherapists while the MIND questionnaire is meant to be a multi-disciplinary tool.

The MIND questionnaire was developed to minimize burden in evaluating PMV patients’ health status by using a short number of items that are easily performed in daily practice. This was confirmed by the professionals participating in the study who indicated that the MIND questionnaire took 10–15 min to complete, and did not alter significantly normal clinical evaluation as all items are generally assessed routinely. For this evaluation only an oximeter and a peak flow meter are needed. So because of its clinical utility, the MIND questionnaire may be adopted as the appropriate test to evaluate patients under PMV.

The MIND questionnaire was included for validation in a multicentre study, including patients from Europe and South-America, recruited from five inpatient facilities. The composition of the sample in terms of disease categories is in line with what can be expected for patients who normally are admitted to weaning facilities [30, 31]. They were tracheotomized patients, almost half with complete ventilator dependence, undergoing very frequent suctioning, unable to stand, having two or more co-morbidities and needing a gastrostomy tube. The centers shared similar characteristics, protocols and staffing, providing identical rehabilitation programs. Therefore, the MIND questionnaire should be tested further in different centers for PMV patients not affiliated to the REMEO® centers for further validity.

The 11 components of the MIND were demonstrated to be relatively independent, confirming the composite nature of the measure. Still upper and lower limb strength and mobility were correlated with Feeding/swallowing suggesting that muscle dysfunction includes not only limb but also swallowing muscles. In fact, oropharyngeal dysphagia and skeletal muscle weakness are common features of PMV [32], probably related to secondary sarcopenia [33]. Moreover secretion encumbrance is highly prevalent in PMV [34] and inability to clear secretions may lead/worsen hypoventilation [35].

Four composite scores were created: the MIND 11-component score (simple summation and weighted), which encompassed all MIND components, and the short-form MIND 2-component score (simple summation and weighted) including only Feeding/Swallowing and Cough strength, which were empirically shown to capture most of the information regarding the overall health status reported by patients. Feeding/swallowing and Cough strength dysfunction are frequently perceived as bothersome for PMV patients [36]. Swallowing dysfunction is a common complication in PMV patients, with impact on clinical outcomes such as delay in weaning and decannulation process [37]. Moreover, it has been shown that cough peak flow predicts pulmonary complications in dysphagic patients [38] and correlates significantly with the CPAx score [13]. In addition, insufficient cough strength has a major role in failed extubation/decannulation in patients with high level spinal cord injury, primary neuromuscular disorders or ICU-acquired weakness [4].

Overall, all MIND composite and component scores showed very good measurement properties: construct validity was supported by their associations with MRC scales, EQ-5D, SOFA, DNS and GCS, and test-retest reliability was good as indicated by coefficients above recommended thresholds for the majority of them. Most scores showed a good ability to detect improvement in patient health status.

However, additional research is required to evaluate the feasibility of using this instrument in other groups of patients, e.g. PMV patients living at home, and to test its responsiveness to multi-disciplinary rehabilitation programs.

We acknowledge that our research is not without limitations. First, in the study the MIND questionnaire was administered in different phases of subject admission (new admissions and existing inpatients), which means that the sample was heterogeneous. Nonetheless, as our objective was the validation of the tool, performing analyses on data that mirrors the patient population of the facilities is probably not a critical issue. Second, even though the study was conducted in a multinational setting, the MIND questionnaire was not translated. Even though this may have impacted the reliability of responses, it was judged that healthcare professionals of the clinics would be able to respond to questions in English. Third, our study design did not allow for the estimation of inter-rater reliability. Fourth, the MIND scores could not detect worsening in patients’ health status in our study. This may be due to the fact that inpatients had poor health status at baseline and the MIND score would not be able to detect worsening in this already severe population. Finally, only the EQ-5D was included to characterize individual perspective in the study. It is, however, one of the most frequently used patient rated quality of life questionnaires in ventilated patients [39] and is also considered an appropriate instrument for measuring the patient perspective in multicentre critical care trials [40]. More specific quality of life questionnaires like the Severe Respiratory Insufficiency Questionnaire-SRI [41, 42] and the recently developed Quality of Life questionnaire for mechanically ventilated ICU patients-QOL-MV [39] may have allowed gain a better understanding of the perspective of PMV patients and how this complements the healthcare professionals’ perspective captured by the MIND questionnaire.

## Conclusion

The MIND questionnaire was specifically designed as a comprehensive assessment of the health status of patients under PMV that could serve for evaluating clinical severity in these settings. It allows the characterization of 11 key aspects of health status for these patients, with a summary score, as well as a short-form 2-component score that summarizes most of the information collected by the MIND questionnaire and therefore could be used in a setting where a shorter instrument is needed.

## Additional file

Additional file 1: MIND questionnaire. (PDF 859 kb)

Additional file 2: Manual for the Completion of the Multi-INdependence Dimensions (MIND) questionnaire. (DOC 88 kb)

Additional file 3: EQ-5D item scores at baseline. (DOCX 18 kb)

Additional file 4: Comparison of MIND component and composite scores at baseline according to MRC scores, SOFA score and GCS. (DOCX 21 kb)

## Data Availability

The datasets used and/or analysed during the current study are available from the corresponding author on reasonable request.

## Abbreviations

BWAP: Burns Wean Assessment Program
CGIC: Clinician global impression of change
CPAx: Chelsea critical care physical assessment tool
DNS: Dependence nursing scale
EQ-5D: EuroQol-5 dimension
FIM: The functional independence measure
FSS-ICU: Functional status score for the intensive care unit
GCS: Glasgow coma scale
ICU: Intensive care unit
MIND: Multi-INdependence Dimensions
MRC: Medical research council
NAMDRC: National Association for Medical Direction of Respiratory Care
PFIT-s: Physical function ICU test
PMV: Prolonged mechanical ventilation
QOL-MV: Quality of Life questionnaire for mechanically ventilated ICU patients
SOFA: Sepsis-related organ failure assessment
SRI: Severe respiratory insufficiency questionnaire
