# Supplementary material for: Validation of the Multi-INdependence Dimensions (MIND) questionnaire for prolonged mechanically ventilated subjects
Source: BMC Pulm Med. 2019 Jun 20;19:109. doi: 10.1186/s12890-019-0870-2 (PMC6585039; doi:10.1186/s12890-019-0870-2)
Supplement: Supplementary file 1 — MIND questionnaire. (PDF 859 kb) [file 12890_2019_870_MOESM1_ESM.pdf]

MIND Scale

Multi-INdependence Dimensions questionnaire

Version April 2019

MIND (I)

1. Cognition (average both items)

Orientation[1]

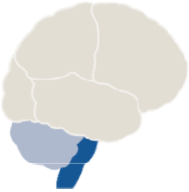

0	1	2	3	4	5
Comatose or vegetative state	Confused, confabulatory, agitated	Disoriented in place	Altered sleep – wake rhythm	Disoriented in time	Oriented, peaceful and cooperative

Speech[2]

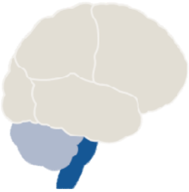

0	1	2	3	4	5
Loss of useful speech (anarthria or not fluently aphasia)	Fluently aphasic	Speech combined with non-verbal communications	Intelligible with repeating	Detectable speech disturbance	Normal

MIND (II)

2. Feeding/swallowing[3]

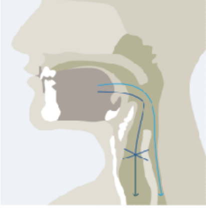

0	1	2	3	4	5
Needs parenteral, or gastrostomy feeding	Partially dependent on non-oral enteral/parenteral feeding. Safe swallowing only when consistency is modified (needs supervision of speech pathologist)	Primary mode of nutrition is oral. Additional time usually necessary for meals because of swallowing strategies	Needs partial assistance for eating and/or drinking, or for wearing adaptive devices	Eats independently; needs adaptive devices or assistance only for cutting food and/or pouring and/or opening containers	Eats and drinks independently; does not require assistance or adaptive devices

MIND (III)

3. Sleep (total sum of 5 questions/5)

Rate the patient's last night:

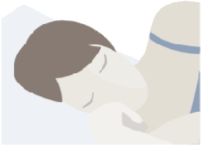

	0	1	2	3	4	5
Sleep depth	Light					Deep
Sleep latency	Very long					Short
Awakenings	Very frequent					Very few
Return to sleep	Very difficult					Very easy
Quality of sleep	Very bad					Very good

MIND(IV)

4. Skin integrity[4]

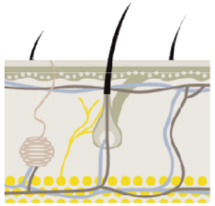

0	1	2	3	4	5
Stage IV: Full thickness tissue loss (muscle/ bone visible) in one or more anatomical locations [a)]	Stage III pressure ulcer (Full thickness skin loss: fat visible) [b)] in one or more anatomical locations	Stage II pressure ulcer (Partial thickness skin loss or blister)[c)] in one or more anatomical location	Stage I pressure ulcer (Non-blanchable redness of intact skin in more than one anatomical location	Stage I pressure ulcer (Non-blanchable redness of intact skin) [d)] in one one anatomical location	Normal

MIND (V-VI)

5. Oxygenation

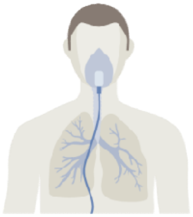

0	1	2	3	4	5
spO_2/FiO_2 ≤235	spO_2/FiO_2 >236≤285	spO_2/FiO_2 >286≤335	spO_2/FiO_2 >336≤385	spO_2/FiO_2 >386 ≤ 435	spO_2/FiO_2 >436

6. Cough strength

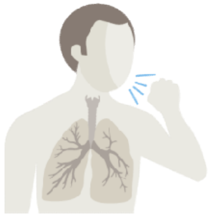

0	1	2	3	4	5
Unable to cough efficiently (PCF ≤ 100L/min)	Very weak voluntary cough (PCF >101 ≤ 160L/min)	Weak voluntary cough (PCF >161 ≤ 220L/min)	Acceptable voluntary cough (PCF >221 ≤ 270L/min)	Effective voluntary cough (PCF >271 ≤ 400L/min)	Normal cough (PCF >401L/min)

MIND (VII)

7. Secretion management

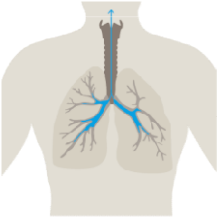

0	1	2	3	4	5
Very frequent deep suctioning* (>8 per day) AND/OR frequent use of in-exsufflator through tracheostomy tube (>8 sessions per day)	Frequent deep suctioning* (8-4 per day) AND/OR frequent use of in-exsufflator through tracheostomy tube (8-4 sessions[5] per day)	Suctioning <4 times per day AND/OR use of in-exsufflator through tracheostomy tube (<4 sessions per day)	Frequent use of non-invasive in-exsufflator (≥4 sessions per day)	Use of non-invasive in-exsufflator (<4 sessions per day)	Clearing secretions independently

MIND (VIII)

8. Mobility (average both items)

Sit to stand[6]

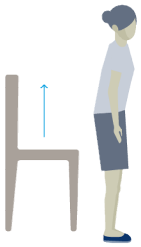

0	1	2	3	4	5
Unable/ unstable	With maximal assistance (standing hoist or similar)	With moderate assistance (eg one or two people)	With minimum assistance (eg one person)	Independently pushing through arms of chair	Independently without upper limb involvement

MIND (VIII)

8. Mobility (average both items)

Stand to sit[7]

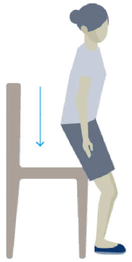

0	1	2	3	4	5
Unable/ unstable	With maximal assistance (two or more people)	With moderate assistance (at least one person)	With minimum assistance (only one person)	Independent with some dynamic sitting balance (ie able to alter trunk position within base of support)	Independent with full dynamic sitting balance (ie able to reach out of base of support)

MIND (IX)

9. Upper limb and lower limb strength (average both items)[8]

Upper limb strength

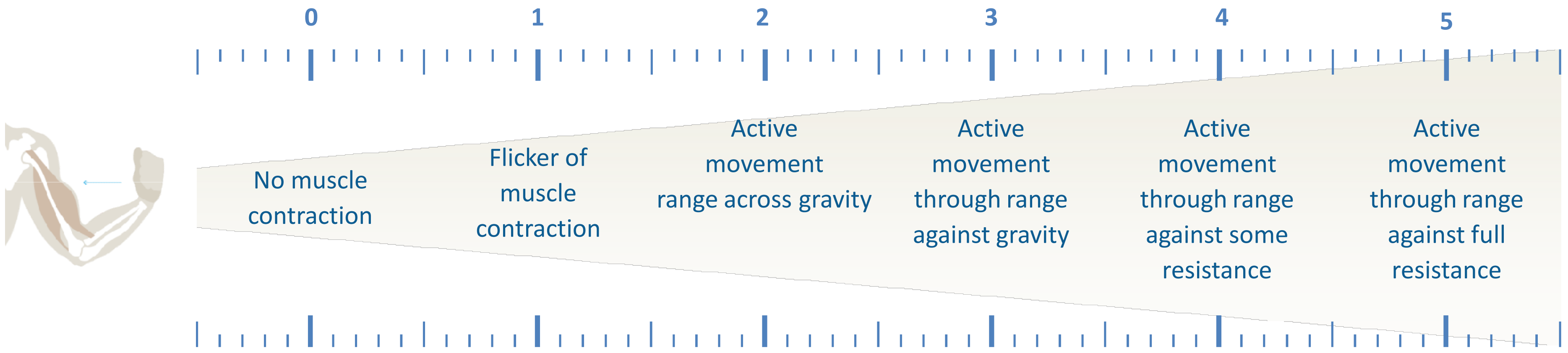

MIND (IX)

9. Upper limb and lower limb strength (average both items)[8]

Lower limb strength

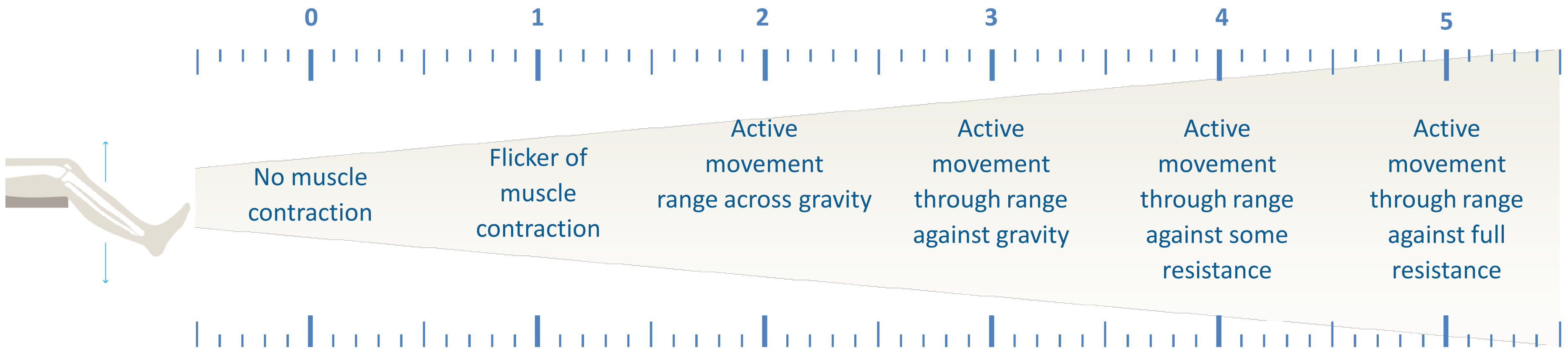

MIND (X)

10. Ventilator dependence[9]

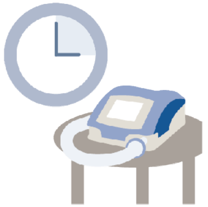

0	1	2	3	4	5
Requires tracheal tube and complete ventilator dependence	Requires tracheal tube and ventilator dependence for most of the day (20min-4h ventilator free-breathing time) OR spontaneous breathing with continuous non-invasive ventilator support (16-24h)	Requires tracheal tube and nocturnal ventilator dependence (12-20h ventilator free-breathing time)	Spontaneous breathing for 24h but requires tracheal tube due to aspiration/bulbar dysfunction	Spontaneous breathing with nocturnal non-invasive ventilator support	Breathes independently without ventilator assistance (or complete ventilator independence)

MIND (XI)

11. Co-morbidities

From the Modified Charlson index list:

1. Congestive heart failure, 2. Coronary artery disease, 3. Diabetes, 4. Peripheral vascular disease, 5. Stroke, 6. Chronic Obstructive Pulmonary disease, 7. Connective tissue disorder, 8. Renal insufficiency, 9. Malignancy

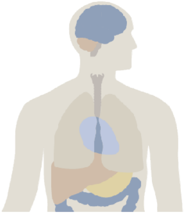

0	1	2	3	4	5
More than three co-morbidities (including one or more of these: diabetes, stroke, renal insufficiency and malignancy)	More than three co-morbidities	Two to three co-morbidities (including one or m ore of these: diabetes, stroke, renal insufficiency and malignancy	Two to three co-morbidities	One co-morbidity	No co-morbidities
