# Supplementary material for: Validation of the Multi-INdependence Dimensions (MIND) questionnaire for prolonged mechanically ventilated subjects
Source: BMC Pulm Med. 2019 Jun 20;19:109. doi: 10.1186/s12890-019-0870-2 (PMC6585039; doi:10.1186/s12890-019-0870-2)
Supplement: Supplementary file 2 — Manual for the Completion of the Multi-INdependence Dimensions (MIND) questionnaire. (DOC 88 kb) [file 12890_2019_870_MOESM2_ESM.doc]

**Manual for the Completion of the Multi-INdependence Dimensions (MIND) questionnaire**

For a complete layout of the questionnaire see below

Definitions:

| **Term** | **Definition** |
| --- | --- |
| **Aphasia** | Loss or impairment of the power to use or comprehend words. |
| **Anarthria** | The loss of the ability to speak words properly. |
| **Non-fluent aphasia' (or Broca's aphasia)** | Speech output is severely reduced and is limited mainly to short utterances of less than four words. Vocabulary access is limited and the formation of sounds by persons with Broca's aphasia is often laborious and clumsy. The person may understand speech relatively well and be able to read, but be limited in writing. |
| **Fluent aphasia ( or Wernicke's aphasia)** | In this form of aphasia the ability to grasp the meaning of spoken words is chiefly impaired, while the ease of producing connected speech is not much affected. Therefore Wernicke's aphasia is referred to as a 'fluent aphasia.' However, speech is far from normal. Sentences do not hang together and irrelevant words intrude-sometimes to the point of jargon, in severe cases. Reading and writing are often severely impaired. |

**Guidance to complete the MIND questionnaire**

**Cognition domain:**

| To test **speech,** make sure that in tracheostomized patients you are using an uncuffed cannula or in case of a cuffed cannula the cuff is deflated and you increase the tidal volume or PEEP level; moreover specify if you are using a speaking valve. |
| --- |

**Sleep** **domain:**

| The nurse completes this section at the end of their night shift. Subjective assessment of sleep depth, sleep latency, sleep disruption (awakenings), return to sleep and quality of sleep was determined using a 0-5 scale |
| --- |

**Skin integrity** domain:

| Assess the following anatomical sites :heels, sacrum, elbow, scapulae, back of the head. See the suggested definitions: |
| --- |
| *Stage IV*-Full thickness tissue loss. Subcutaneous fat may be visible but bone, tendon or muscle are not exposed. Some slough may be present. May include undermining and tunneling. |
| The depth of a Category/*Stage III* pressure ulcer varies by anatomical location. The bridge of the nose, ear, occiput and malleolus do not have (adipose) subcutaneous tissue and Category/Stage III ulcers can be shallow. In contrast, areas of significant adiposity can develop extremely deep Category/Stage III pressure ulcers. Bone/tendon is not visible or directly palpable |
| *Stage II*-Partial thickness loss of dermis presenting as a shallow open ulcer with a red pink wound bed, without slough. May also present as an intact or open/ruptured serum-filled or sero-sanguineous filled blister. Further description: Presents as a shiny or dry shallow ulcer without slough or bruising. This category/stage should not be used to describe skin tears, tape burns, incontinence associated dermatitis, maceration or excoriation. |
| *Stage I*-Intact skin with non-blanchable erythema of a localized area usually over a bony prominence. Discoloration of the skin, warmth, edema, hardness or pain may also be present. Darkly pigmented skin may not have visible blanching. Further description: The area may be painful, firm, soft, warmer or cooler as compared to adjacent tissue. Category/Stage I may be difficult to detect in individuals with dark skin tones. May indicate “at risk” persons. |

Oxygenation

| To calculate FiO2 use Oxygen sensor (external or included in the ventilator) or Normogram |
| --- |
| 0 spO2/FiO2 <235 |
| 1 spO2/FiO2 ≥235<285 |
| 2 spO2/FiO2 ≥285<335 |
| 3 spO2/FiO2 ≥335<385 |
| 4 spO2/FiO2 ≥385<435 |
| 5 spO2/FiO2 ≥435 |

Evaluation of **cough strength**

| To undertake this evaluation position the patient supine with the head of the bed elevated at 30º. Deflate the cuff (make sure you suction above the cuff before the cuff is deflated). With a Passy-Muir valve or cap applied to the tracheostomy, instruct the patient to cough as forcefully as possible, measuring the Cough Peak Flow rate-CPFR (with a peak flow meter attached to a transparent ambu bag mask) at the mouth. In case you measure the peak flow through the tracheostomy tube, attach a connector linked to a viral/bacterial respiratory filter and the Electronic Peak Flow Meter (PiKo1) and proceed in the same way. Select the best of 3 trials. Oxygen saturation and signs of respiratory distress should be monitored during the measurement process. The CPFR measurement procedure should be stopped immediately if any of the following occur: respiratory rate, greater than 35/min; oxygen saturation as measured by pulse oximetry, less than 90%; heart rate, greater than 140/min or an increase of more than 20% above resting levels |
| --- |
| 0 Unable to cough efficiently (PCF<100L/min) |
| 1 Very weak voluntary cough (PCF≥100<160L/min) |
| 2 Weak voluntary cough (PCF≥160<220L/min) |
| 3 Acceptable voluntary cough (PCF≥220<270L/min) |
| 4 Effective voluntary cough (PCF≥270L/min) |
| 5 Normal cough (PCF≥400L/min) |

**.Secretion management**

| Classify secretion status by counting the number of tracheal tube suctioning sessions or tracheal tube or mask in-exsufflation  Ensure that each mechanical cough assistance cycle is: an insufflation/exsufflation time ratio of 3:2 seconds and a pause of 3 seconds between. Every session is eight cycles. |
| --- |
| 0 Very Frequent deep suctioning * (>8 per day) AND/OR frequent use of in-exsufflator through tracheostomy tube (>8 sessions per day) |
| 1 Frequent deep suctioning* (8-4 per day) AND/OR frequent use of in-exsufflator through tracheostomy tube (8-4 sessions5 per day) |
| 2 Suctioning <4 times per day AND/OR use of in-exsufflator through tracheostomy tube (<4 sessions per day) |
| 3 Frequent use of non invasive in-exsufflator (>4 sessions per day) |
| 4 Use of non invasive in-exsufflator <4 sessions per day) |
| 5 Clearing secretions independently |

**Mobility** evaluation:

| **Sit to stand**:  During the evaluation the patient should be seated on supporting surface. He should be asked to come to the standing position |
| --- |
| **Stand to sit:**  During the evaluation the patient should be standing with their back to the supporting surface. He should be asked to sit. |

**Upper and lower limb strength (for more detailed description see also MRC testing instructions below)**:

| Once the patient has returned to the sitting position, bilateral shoulder flexion and bilateral knee extension strength testing should be performed once the patient has had ample time to rest. If the patient is unable to sit out of bed, muscle strength testing should be performed with the patient in supine. For shoulder flexion strength testing, the patient must be sat as upright as possible.  Bilateral shoulder flexion should be performed first. Free active movement should be assisted first, and then resistance applied through range, resisted at the elbow where resistance is required. Muscle strength should be recorded for each arm.  Knee extension should be resisted at the ankle where resistance is required for assessment. Muscle strength should be recorded for each leg. If being performed in supine, inner range knee extension will be assessed by quads over fulcrum with resistance applied at the ankle as required. |
| --- |

**Ventilator Dependence**

| Evaluate ventilator dependence based on free breathing time and spontaneous breathing or tracheostomy tube need |
| --- |
| 0 Requires tracheal tube and complete ventilator dependence |
| 1 Requires tracheal tube and ventilator dependence for most of the day (20min-4h ventilator free-breathing time) OR Spontaneous breathing with continuous non-invasive ventilator support (16-24h) |
| 2 Requires tracheal tube and nocturnal ventilator dependence (12-20h ventilator free-breathing time) |
| 3 Spontaneous breathing for 24h but requires tracheal tube due to aspiration/bulbar dysfunction |
| 4 Spontaneous breathing with nocturnal non-invasive ventilator support |
| 5 Breathes independently without ventilator assistance (or complete ventilator independence) |

**Co-Morbidities**

| 1)Congestive heart failure—documented decreased left ventricular function or mean pulmonary artery pressure >25 mm Hg as determined by stress echocardiography, including patients with portopulmonary hypertension. 2) Coronary artery disease—documented history of myocardial infarction, or coronary disease on angiography. All men above age 40 yr and all women above age 50 yr, as well as patients of any age with risk factors for coronary artery disease underwent a stress test. Patients with a positive stress test but negative angiography were not considered as having coronary artery disease. 3) Diabetes mellitus—chronic hyperglycemia requiring outpatient medications at any time during the month preceding evaluation. 4) Peripheral vascular disease— documented arterial disease by angiography or ankle- brachial index. 5) Cerebral vascular accident—history of stroke with residual neurological deficit. 6) Chronic obstructive pulmonary disease (COPD)—chronic lung disease with requirement for medications, documented forced expiratory volume in 1 second <1.5 L, or a history of intubation for respiratory failure. 7) Connective tissue disease—diagnosis by a rheumatologist of systemic lupus, rheumatoid arthritis, scleroderma, or seronegative spondyloarthropathy. Patients with osteoarthritis, or arthralgias without objective evidence of inflammatory arthritis, were not considered as having connective tissue disease. 8) Renal insufficiency—serum creatinine of 1.5 mg/dL or greater on most recent testing, or a history of renal transplantation. 9) Malignancy—history of malignancy, excluding nonmelanoma skin cancer and hepatocellular carcinoma. |
| --- |

**MRC testing** (Video of MRC testing- (go to : <http://www.ncbi.nlm.nih.gov/pmc/articles/PMC3169254/> and download video: jove-50-2632.mov)

### Biceps

| 1.Test position - forearm supinated and flexed slightly more than 90 degrees. Verbal instructions: "*Bend your elbow slightly more than 90 degrees*". The hand giving resistance is contoured over the flexor surface of the forearm proximal to the wrist. The examiner's other hand applies counterforce by cupping the palm over the anterior superior aspect of the shoulder. The examiner then states: "*Hold it. Don't let me push it down*" and scores Grades 3, 4, or 5 as previously described. |
| --- |
| 2.If weaker than Grade 3, the shoulder is abducted to 90 degrees. The examiner supports the arm under the elbow and, if necessary, the wrist as well. The forearm is turned with the thumb facing the ceiling. With the elbow extended, the patient attempts to flex the elbow. The examiner states: "*Try to bend your elbow.*" Grade 2 is assigned if the patient can flex the elbow. |
| 3.If weaker than Grade 2, the forearm is supinated and positioned at the side in approximately 45 degrees of elbow flexion. The examiner states "*Try to bend your elbow*", palpates the biceps tendon and scores as either Grade 1 or 0. |

### Quadriceps

| 1. Test position - sitting upright with the knee fully extended to 0 degrees. Avoid knee hyperextension. Verbal instructions; "Straighten your knee ". The hand giving resistance is contoured on top of the leg just proximal to the ankle. The other hand is placed under the thigh above the knee. The examiner then states "Hold it. Don't let me bend it" and scores Grades 3, 4 or 5. |
| --- |
| 2. If weaker than Grade 3, the patient lays on the non-testing side. The examiner stands behind the patient at knee level. The leg not being tested may be flexed for stability. One arm cradles the leg being tested around the thigh with the hand supporting the underside of the knee. The other hand holds the leg just above the ankle. The examiner states: "Straighten your knee." Grade 2 is assigned if the patient can extend the knee |
| 3. If weaker than Grade 2, the patient is supine and the examiner states:"Push the back of your knee down" or "Tighten your knee cap" and palpates the quadriceps tendon, and scores as Grade 1 or 0. |
| 4. For the bedridden patient, in scoring Grades 3, 4, and 5, the patient is positioned in the same manner as for hip flexion and graded as described above for knee extension |

| Each muscle group (quadriceps and biceps) is graded as follows: |
| --- |
| 0 - no movement |
| 1 - flicker is perceptible in the muscle |
| 2 - movement only if gravity eliminated |
| 3 - can move limb against gravity |
| 4 - can move against gravity & some resistance exerted by examiner |
| 5 - normal power |
