# Supplementary material for: Validation of the Multi-INdependence Dimensions (MIND) questionnaire for prolonged mechanically ventilated subjects
Source: BMC Pulm Med. 2019 Jun 20;19:109. doi: 10.1186/s12890-019-0870-2 (PMC6585039; doi:10.1186/s12890-019-0870-2)
Supplement: Supplementary file 3 — EQ-5D item scores at baseline. (DOCX 18 kb) [file 12890_2019_870_MOESM3_ESM.docx]

**Supplementary material**

**Additional file 3: Table S1. EQ-5D item scores at baseline (N=128)**

|  |  | **Total (N=128)** |
| --- | --- | --- |
| Mobility item (%) | No problem | 4.7 |
|  | Some problems | 28.9 |
|  | Confined to bed | 52.3 |
|  | Missing | 14.1 |
| Self-care item (%) | No problem | 3.1 |
|  | Some problems | 33.6 |
|  | Unable to wash or dress myself | 49.2 |
|  | Missing | 14.1 |
| Usual activities item (%) | No problem | 3.1 |
|  | Some problems | 28.9 |
|  | Unable to perform usual activities | 52.3 |
|  | Missing | 15.6 |
| Pain/Discomfort item (%) | No pain or discomfort | 35.9 |
|  | Some pain or discomfort | 35.9 |
|  | Extreme pain or discomfort | 14.1 |
|  | Missing | 14.1 |
| Anxiety/Depression item (%) | Not anxious or depressed | 32.8 |
|  | Moderately anxious or depressed | 43.0 |
|  | Extremely anxious or depressed | 10.2 |
|  | Missing | 14.1 |
