# Supplementary material for: Validation of the Multi-INdependence Dimensions (MIND) questionnaire for prolonged mechanically ventilated subjects
Source: BMC Pulm Med. 2019 Jun 20;19:109. doi: 10.1186/s12890-019-0870-2 (PMC6585039; doi:10.1186/s12890-019-0870-2)
Supplement: Supplementary file 4 — Comparison of MIND component and composite scores at baseline according to MRC scores, SOFA score and GCS. (DOCX 21 kb) [file 12890_2019_870_MOESM4_ESM.docx]

**Table S2 Comparison of MIND component and composite scores at baseline according to MRC scores, SOFA score and GCS (N=128)**

| **MIND score^a^** | | **MRC scale quadriceps** | | | | **MRC scale biceps** | | | | **SOFA score** | | | **GCS** | | | |
| --- | --- | --- | --- | --- | --- | --- | --- | --- | --- | --- | --- | --- | --- | --- | --- | --- |
|  |  | **0-2 (N=62)** | **3 (N=26)** | **4-5 (N=37)** | **p-value** | **0-2 (N=46)** | **3 (N=35)** | **4-5 (N=44)** | **p-value** | **0-2 (N=107)** | **3-8 (N=21)** | **p-value** | **1-8 Severe Brain Injury (N=12)** | **9-12 Moderate Injury (N=17)** | **13-15 Mild Injury (N=99)** | **p-value** |
| Component | Cognition | 2.8 | 4.2 | 4.8 | <0.001 | 2.5 | 3.8 | 4.7 | <0.001 | 3.9 | 1.8 | <0.001 | 0.3 | 1.4 | 4.4 | <0.001 |
|  | Feeding/swallowing | 0.9 | 2.2 | 4.2 | <0.001 | 0.9 | 1.6 | 3.8 | <0.001 | 2.5 | 0.2 | <0.001 | 0.0 | 0.1 | 2.7 | <0.001 |
|  | Sleep | 3.2 | 3.2 | 3.3 | 0.793 | 3.3 | 3.1 | 3.3 | 0.464 | 3.3 | 2.9 | 0.194 | 3.2 | 3.1 | 3.2 | 0.677 |
|  | Skin integrity | 3.9 | 3.9 | 4.8 | 0.005 | 3.9 | 3.9 | 4.6 | 0.027 | 4.2 | 3.9 | 0.307 | 4.8 | 3.8 | 4.2 | 0.202 |
|  | Oxygenation | 2.9 | 2.7 | 2.6 | 0.665 | 3.0 | 2.9 | 2.4 | 0.143 | 2.7 | 2.9 | 0.642 | 2.5 | 3.2 | 2.7 | 0.337 |
|  | Cough strength | 1.5 | 1.9 | 1.4 | 0.533 | 1.4 | 1.9 | 1.4 | 0.337 | 1.4 | 2.0 | 0.131 | 1.0 | 1.6 | 1.6 | 0.548 |
|  | Secretion management | 1.6 | 1.5 | 1.7 | 0.888 | 1.7 | 1.7 | 1.5 | 0.697 | 1.6 | 1.4 | 0.515 | 1.3 | 1.8 | 1.6 | 0.588 |
|  | Mobility | 0.1 | 1.7 | 3.2 | <0.001 | 0.2 | 1.1 | 2.8 | <0.001 | 1.5 | 0.2 | 0.001 | 0.0 | 0.1 | 1.7 | <0.001 |
|  | Upper limb and lower limb strength | 1.4 | 3.4 | 4.4 | <0.001 | 1.1 | 2.8 | 4.3 | <0.001 | 2.9 | 1.6 | 0.001 | 0.5 | 1.7 | 3.1 | <0.001 |
|  | Ventilator dependence | 1.1 | 0.9 | 2.1 | 0.001 | 1.1 | 1.3 | 1.7 | 0.121 | 1.3 | 1.2 | 0.792 | 1.3 | 1.1 | 1.4 | 0.736 |
|  | Co-morbidities | 3.3 | 2.3 | 2.8 | 0.015 | 3.5 | 2.6 | 2.6 | 0.003 | 2.9 | 3.1 | 0.547 | 3.6 | 2.9 | 2.8 | 0.268 |
| 11-component composite | Simple summation | 22.6 | 27.6 | 35.3 | <0.001 | 22.5 | 26.6 | 33.2 | <0.001 | 28.2 | 21.3 | <0.001 | 18.2 | 20.7 | 29.3 | <0.001 |
|  | Weighted summation | 35.8 | 55.3 | 72.7 | <0.001 | 34.2 | 49.2 | 69.4 | <0.001 | 53.5 | 32.4 | <0.001 | 21.0 | 28.9 | 57.1 | <0.001 |
| 2-component composite | Simple summation | 2.4 | 4.0 | 5.5 | <0.001 | 2.3 | 3.5 | 5.3 | <0.001 | 3.9 | 2.2 | 0.012 | 1.0 | 1.7 | 4.2 | <0.001 |
|  | Weighted summation | 7.8 | 12.5 | 16.5 | <0.001 | 7.3 | 11.2 | 15.8 | <0.001 | 11.8 | 7.7 | 0.039 | 3.5 | 5.9 | 12.9 | <0.001 |
| MRC = Medical Research Council; SOFA = Sepsis-related Organ Failure Assessment; GCS = Glasgow Coma Scale  ^a^Mean MIND score for each group with p-value from t-test or ANOVA | | | | | | | | | | | | | | | | |
